# Supplementary material for: MetaRibo-Seq measures translation in microbiomes
Source: Nat Commun. 2020 Jun 29;11:3268. doi: 10.1038/s41467-020-17081-z (PMC7324362; doi:10.1038/s41467-020-17081-z)
Supplement: Supplementary file 10 — Supplementary Data 7 [file 41467_2020_17081_MOESM10_ESM.zip › File2/Confidence_VeryHigh_Taxonomy/62392_out.krona.html]

Javascript must be enabled to view this page.

members
magnitude
magnitudeUnassigned
count
unassigned
taxon
rank

62392\_out

54

2
54
superkingdom

phylum
54
976

54
200643
class

171549
54
order

family
815
1

816
1

SRS016095\_contig\_number\_12775
genus

family
53
171552

53
577309
genus

454155
5

SRS013687\_contig\_number\_27827SRS044535\_contig\_number\_18463SRS077641\_contig\_number\_19076SRS1041134\_contig\_number\_5614SRS146764\_contig\_number\_8659
species

17
1263095
species

SRS013800\_contig\_number\_13394SRS014534\_contig\_number\_contig-100\_49.50SRS017701\_contig\_number\_3616SRS023914\_contig\_number\_21080SRS024435\_contig\_number\_21987SRS047433\_contig\_number\_13339SRS048164\_contig\_number\_28068SRS049712\_contig\_number\_contig-100\_3038.71803SRS050026\_contig\_number\_9543SRS050520\_contig\_number\_contig-100\_2810.2811SRS053649\_contig\_number\_9987SRS063985\_contig\_number\_28032SRS078665\_contig\_number\_8123SRS1041037\_contig\_number\_9700SRS1041130\_contig\_number\_4924SRS893187\_contig\_number\_contig-100\_13702.13703SRS893366\_contig\_number\_20250


SRS015782\_contig\_number\_26463SRS016381\_contig\_number\_2SRS017307\_contig\_number\_19434SRS017433\_contig\_number\_2907SRS017821\_contig\_number\_18586SRS024492\_contig\_number\_contig-100\_397.74728SRS043841\_contig\_number\_2144SRS045004\_contig\_number\_25042SRS045645\_contig\_number\_contig-100\_2440.107578SRS047044\_contig\_number\_12123SRS049896\_contig\_number\_12126SRS049995\_contig\_number\_15508SRS055017\_contig\_number\_contig-100\_7617.143638SRS057480\_contig\_number\_contig-100\_38200.38201SRS063370\_contig\_number\_8513SRS064232\_contig\_number\_contig-100\_11347.11348SRS077294\_contig\_number\_11048SRS077589\_contig\_number\_325SRS078176\_contig\_number\_17300SRS101376\_contig\_number\_25652SRS1041031\_contig\_number\_27027SRS1041116\_contig\_number\_665SRS1041118\_contig\_number\_3093SRS1041138\_contig\_number\_9248SRS1041140\_contig\_number\_11926SRS105082\_contig\_number\_3504SRS143070\_contig\_number\_15301SRS143181\_contig\_number\_contig-100\_36765.36765SRS143876\_contig\_number\_17236SRS147653\_contig\_number\_2712SRS893252\_contig\_number\_16838
species
454154
31
